# Supplementary material for: Asynchronous magnetic resonance elastography: Shear wave speed reconstruction using noise correlation of incoherent waves
Source: Magn Reson Med. 2022 Oct 27;89(3):990–1001. doi: 10.1002/mrm.29502 (PMC9792433; doi:10.1002/mrm.29502)
Supplement: Supplementary file 1 — DATA S1 MATLAB code used to generate the numerical simulations of Figure 1 [file MRM-89-990-s002.zip › k-Wave/helpfiles/example_ivp_1D_simulation.html]

Simulations In One Dimension Example (k-Wave)


# Simulations In One Dimension Example

This example provides a simple demonstration of using k-Wave for the simulation and detection of the pressure field generated by an initial pressure distribution within a one-dimensional heterogeneous propagation medium. It builds on the Homogeneous Propagation Medium and Heterogeneous Propagation Medium examples.

- Open the file in the MATLAB Editor
- Run the file in MATLAB

## Contents

- Creating the k-space grid and defining the medium properties
- Defining the initial pressure distribution and sensor mask
- Running the simulation

## Creating the k-space grid and defining the medium properties

Simulations in one-dimension are performed in an analogous fashion to those in two-dimensions. The medium discretisation is again performed by `kWaveGrid` using the inputs for a single dimension. The properties of a heterogeneous acoustic propagation medium are also given as one-dimensional column vectors.

```
% create the computational grid
Nx = 512;       % number of grid points in the x (row) direction
dx = 0.05e-3;   % grid point spacing in the x direction [m]
kgrid = kWaveGrid(Nx, dx);

% define the properties of the propagation medium
medium.sound_speed = 1500 * ones(Nx, 1);    % [m/s]
medium.sound_speed(1:round(Nx/3)) = 2000;   % [m/s]
medium.density = 1000 * ones(Nx, 1);        % [kg/m^3]
medium.density(round(4*Nx/5):end) = 1500;   % [kg/m^3]
```

## Defining the initial pressure distribution and sensor mask

As in two-dimensions, the initial pressure distribution is set using a vector which contains the initial pressure values for each grid point within the computational domain. Here a smoothly varying pressure function is defined using a portion of a sinusoid.

```
% create initial pressure distribution using a smoothly shaped sinusoid
x_pos = 280;    % [grid points]
width = 100;    % [grid points]
height = 1;     % [Pa]
in = (0:pi/(width/2):2*pi).';
source.p0 = [zeros(x_pos, 1); ((height/2) * sin(in - pi/2) + (height/2)); zeros(Nx - x_pos  - width - 1, 1)];
```

Again the sensor mask, which defines the locations where the pressure field is recorded at each time-step, can be given as a list of Cartesian coordinates, a binary mask, or the grid coordinates of two opposing ends of a line. In this example, a Cartesian sensor mask with two points is defined.

```
% create a Cartesian sensor mask
sensor.mask = [-10e-3, 10e-3];  % [mm]
```

## Running the simulation

The computation is started by passing the four input structures, `kgrid`, `medium`, `source`, and `sensor` to `kspaceFirstOrder1D`. To record long enough to capture the reflections from the heterogeneous interfaces, the time sampling is defined using the `makeTime` method of the `kWaveGrid` class. By default, a visualisation of the propagating wave-field and a status bar are displayed.

```
% set the simulation time to capture the reflections
t_end = 2.5 * kgrid.x_size / max(medium.sound_speed(:));

% define the time array
kgrid.makeTime(medium.sound_speed, [], t_end);

% run the simulation
sensor_data = kspaceFirstOrder1D(kgrid, medium, source, sensor, 'PlotLayout', true);
```

As the function runs, status updates and computational parameters are printed to the command line.

```
Running k-Wave simulation...
  start time: 13-Apr-2017 21:49:35
  reference sound speed: 2000m/s
  dt: 7.5ns, t_end: 31.995us, time steps: 4267
  input grid size: 512 grid points (25.6mm)
  maximum supported frequency: 15MHz
  smoothing p0 distribution...
  precomputation completed in 0.53842s
  starting time loop...
  estimated simulation time 4.011s...
  simulation completed in 4.0106s
  total computation time 4.618s
```

A plot of the initial pressure distribution, sensor mask, and medium properties (returned using `'PlotLayout'` set to `true`) is given below.

When the time loop has completed, the function returns the recorded time series at each of sensor points defined by `sensor_mask`. The ordering is again dependent on whether a Cartesian or binary sensor mask is used. A visualisation of the recorded sensor data is given below.

Note, in some cases the animated visualisation can significantly slow down the simulation. The computational speed can be increased by switching off the animations by setting the optional input `'PlotSim'` to `false`.
